# High-Sensitivity Cardiac Troponin and the Risk Stratification of Patients With Renal Impairment Presenting With Suspected Acute Coronary Syndrome

**DOI:** 10.1161/CIRCULATIONAHA.117.030320

**Published:** 2018-01-29

**Authors:** Eve Miller-Hodges, Atul Anand, Anoop S.V. Shah, Andrew R. Chapman, Peter Gallacher, Kuan Ken Lee, Tariq Farrah, Nynke Halbesma, James P. Blackmur, David E. Newby, Nicholas L. Mills, Neeraj Dhaun

**Affiliations:** 1University/British Heart Foundation Centre for Cardiovascular Science, University of Edinburgh, Queen’s Medical Research Institute, United Kingdom (E.M.-H., A.A., A.S.V.S., A.R.C., P.G., K.K.L., T.F., J.P.B., D.E.N., N.L.M., N.D.); 2Department of Renal Medicine, Royal Infirmary of Edinburgh, United Kingdom (E.M.-H., P.G., T.F., N.D.); 3Usher Institute of Population Health Sciences and Informatics, University of Edinburgh, United Kingdom (N.H.)

**Keywords:** acute coronary syndrome, myocardial infarction, renal insufficiency, risk, troponin

## Abstract

**Background:**

High-sensitivity cardiac troponin testing may improve the risk stratification and diagnosis of myocardial infarction, but concentrations can be challenging to interpret in patients with renal impairment, and the effectiveness of testing in this group is uncertain.

**Methods:**

In a prospective multicenter study of consecutive patients with suspected acute coronary syndrome, we evaluated the performance of high-sensitivity cardiac troponin I in those with and without renal impairment (estimated glomerular filtration rate <60mL/min/1.73m^2^). The negative predictive value and sensitivity of troponin concentrations below the risk stratification threshold (5 ng/L) at presentation were reported for a primary outcome of index type 1 myocardial infarction, or type 1 myocardial infarction or cardiac death at 30 days. The positive predictive value and specificity at the 99th centile diagnostic threshold (16 ng/L in women, 34 ng/L in men) was determined for index type 1 myocardial infarction. Subsequent type 1 myocardial infarction and cardiac death were reported at 1 year.

**Results:**

Of 4726 patients identified, 904 (19%) had renal impairment. Troponin concentrations <5 ng/L at presentation identified 17% of patients with renal impairment as low risk for the primary outcome (negative predictive value, 98.4%; 95% confidence interval [CI], 96.0%–99.7%; sensitivity 98.9%; 95%CI, 97.5%–99.9%), in comparison with 56% without renal impairment (*P*<0.001) with similar performance (negative predictive value, 99.7%; 95% CI, 99.4%–99.9%; sensitivity 98.4%; 95% CI, 97.2%–99.4%). The positive predictive value and specificity at the 99th centile were lower in patients with renal impairment at 50.0% (95% CI, 45.2%–54.8%) and 70.9% (95% CI, 67.5%–74.2%), respectively, in comparison with 62.4% (95% CI, 58.8%–65.9%) and 92.1% (95% CI, 91.2%–93.0%) in those without. At 1 year, patients with troponin concentrations >99th centile and renal impairment were at greater risk of subsequent myocardial infarction or cardiac death than those with normal renal function (24% versus 10%; adjusted hazard ratio, 2.19; 95% CI, 1.54–3.11).

**Conclusions:**

In suspected acute coronary syndrome, high-sensitivity cardiac troponin identified fewer patients with renal impairment as low risk and more as high risk, but with lower specificity for type 1 myocardial infarction. Irrespective of diagnosis, patients with renal impairment and elevated cardiac troponin concentrations had a 2-fold greater risk of a major cardiac event than those with normal renal function, and should be considered for further investigation and treatment.

**Clinical Trial Registration:**

URL: https://www.clinicaltrials.gov. Unique identifier: NCT01852123.

## Abstract 2

Supplemental Digital Content is available in the text.

**Editorial, see p 452**

**Clinical Perspective**

**What Is New?**

- This is the first study to evaluate high-sensitivity cardiac troponin I testing in unselected, consecutive patients with suspected acute coronary syndrome with and without renal impairment.
- Patients with troponin concentrations <5 ng/L at presentation were low risk for myocardial infarction or cardiac death regardless of renal function, but only 1 in 5 patients with renal impairment were identified as low risk.
- Patients with renal impairment were twice as likely to have troponin concentrations >99th centile, with lower specificity for type 1 myocardial infarction, but, irrespective of the diagnosis, these patients had a 2-fold greater risk of cardiac events at 1 year.

**What Are the Clinical Implications?**

- Our findings support the use of high-sensitivity cardiac troponin I testing, using a risk stratification threshold of <5 ng/L, to rule out myocardial infarction in patients with renal impairment.
- The use of diagnostic thresholds above the 99th centile might improve specificity for type 1 myocardial infarction in patients with renal impairment.
- However, such strategies may falsely reassure clinicians that patients below this threshold are at low risk.
- High-sensitivity cardiac troponin I has major potential to risk stratify patients with renal impairment and suspected acute coronary syndrome.

Cardiovascular disease is the most frequent outcome of chronic kidney disease.^1^ As glomerular filtration rate (GFR) declines, major adverse cardiovascular events, cardiovascular disease, and all-cause mortality increase.^1–3^ In patients with acute coronary syndrome, renal impairment is common^4^ and is associated with an increased risk of recurrent myocardial infarction and death.^5,6^ Cardiac troponin testing is used widely to diagnose myocardial infarction,^7–10^ but levels can be challenging to interpret in patients with renal impairment.^11,12^ Circulating troponin concentrations are often raised in these patients because of shared risk factors and preexisting cardiovascular disease.^13^ High-sensitivity cardiac troponin assays permit the use of lower thresholds to rule in and rule out myocardial infarction,^14^ but the effectiveness of testing in patients with renal impairment is uncertain.

We have previously evaluated high-sensitivity cardiac troponin testing in consecutive patients with suspected acute coronary syndrome and defined thresholds for risk stratification (<5 ng/L) and diagnosis of myocardial infarction using sex-specific 99th centile upper reference limits.^7,15^ Patients with cardiac troponin concentrations <5 ng/L at presentation were at low risk of future cardiac events and may not require serial testing or hospital admission.^7,16,17^ The use of sex-specific diagnostic thresholds identified more women with myocardial infarction who were at an increased risk of major cardiac events.^14^ The benefits of both approaches may be offset in patients with comorbid conditions and especially those with renal impairment, where myocardial injury often occurs without acute coronary syndrome, and where high-sensitivity cardiac troponin testing may contribute to diagnostic uncertainty. We aimed to evaluate the performance of high-sensitivity cardiac troponin I testing in consecutive patients with suspected acute coronary syndrome with and without renal impairment.

## Methods

### Study Design and Participants

In a prospective multicenter study, we identified consecutive patients presenting with suspected acute coronary syndrome to the emergency departments of 2 secondary care hospitals (St John’s Hospital, Livingston, and Western General Hospital, Edinburgh) and a tertiary care hospital (Royal Infirmary of Edinburgh) between June 1, 2013, and January 31, 2014.^7^ All patients in whom the attending clinician requested cardiac troponin for suspected acute coronary syndrome were enrolled. Patients were excluded if they were diagnosed with ST-segment–elevation myocardial infarction, had been admitted previously during the study period, or did not live in Scotland and therefore could not have hospital records linked with outcomes. In this analysis, we identified those patients who also had at least 1 measurement of serum creatinine during the index presentation. The study was approved by the national research ethics committee, and performed in accordance with the Declaration of Helsinki. Informed consent was not required.

### Procedures

Plasma cardiac troponin I concentration was measured at presentation and then repeated 6 or 12 hours after the onset of symptoms at the discretion of the clinician. All patients who met the inclusion criteria were assigned a study code and additional data from the electronic patient record (TrakCare; InterSystems Corporation) were collected prospectively and linked in real time with a unique patient identifier.

Clinical decision making used a validated standard-of-care sensitive cardiac troponin I assay (ARCHITECT_*STAT*_ troponin I assay; Abbott Laboratories).^18,19^ High-sensitivity cardiac troponin I was measured in parallel on excess plasma in all enrolled patients, at all time points, using a high-sensitivity assay (ARCHITECT_*STAT*_ high-sensitive troponin I assay; Abbott Laboratories). These results were not reported on the electronic patient record or communicated to the clinicians responsible for patients’ care. For this assay, the limit of detection is 1.2 ng/L with an upper reference limit at the 99th centile in women of 16 ng/L and in men of 34 ng/L.^15,20^ It has a coefficient of variation of 23% at the limit of detection (1.2 ng/L) and <10% at 6 ng/L.^7,21,22^

Baseline clinical characteristics and serum biochemistry results were collected using the electronic patient record. Serum creatinine at presentation was used to calculate the estimated glomerular filtration rate (eGFR) using the Modification of Diet in Renal Disease study equation.^23^ Based on this, patients were classified as having normal (eGFR ≥60mL/min/1.73m^2^) or impaired renal function (eGFR <60mL/min/1.73m^2^). Those with renal impairment were further categorized as having moderate (eGFR 30–59mL/min/1.73m^2^), severe (<30mL/min/1.73m^2^), or end-stage (<15 mL/min/1.73m^2^) renal disease.

Patients with evidence of myocardial necrosis at presentation or on subsequent testing were identified using sex-specific upper reference limits (troponin concentration >99th centile). When patients had serial samples tested, peak troponin was defined as the highest cardiac troponin concentration obtained within 24 hours of hospital presentation. All investigations, clinical information, and outcomes from presentation to 30 days were independently reviewed by 2 adjudicators (A.S. and A.A.). Patients were classified as having type 1 or type 2 myocardial infarction or myocardial injury, according to the Universal Definition of Myocardial Infarction (Table I in the online-only Data Supplement).^11,24^ Type 1 myocardial infarction was defined in patients with myocardial necrosis and symptoms suggestive of acute coronary syndrome or evidence of myocardial ischemia on an ECG. Type 2 myocardial infarction was diagnosed in those patients with symptoms or signs of myocardial ischemia attributed to increased oxygen demand or decreased supply (eg, tachyarrhythmia, hypotension, or anemia). Myocardial injury was defined as biochemical evidence of myocardial necrosis in the absence of any clinical features of myocardial ischemia. Any discrepancies were resolved by the adjudication of a third independent reviewer (N.L.M.). Index myocardial infarction was defined as any type 1 myocardial infarction arising during the first clinical episode. Agreement was good across all adjudicated diagnoses in patients with and without renal impairment (κ 0.72; 95% confidence interval [CI], 0.67–0.78 versus κ 0.70; 95% CI, 0.65–0.75).

Follow-up was completed by using regional and national registries, and the electronic patient record (TrakCare), as well. The same adjudication process as the index admission was used to adjudicate any readmission with elevated cardiac troponin (>99th centile). Cardiac death was defined as any death attributed to myocardial infarction, arrhythmia, or heart failure.

### Outcomes

The negative predictive value (NPV) and sensitivity of cardiac troponin concentrations below the risk stratification threshold (5 ng/L) at presentation were reported for a primary outcome of index type 1 myocardial infarction, or type 1 myocardial infarction or cardiac death at 30 days, as previously described.^7^ We performed an additional sensitivity analysis evaluating the limit of detection as an alternative to this risk stratification threshold. The positive predictive value (PPV) and specificity of cardiac troponin concentrations >99th centile (16 ng/L in women, 34 ng/L in men) on the presentation sample or subsequent testing was determined for index type 1 myocardial infarction. Subgroup analyses were performed stratified by age and sex. In those undergoing serial sampling, performance of the diagnostic threshold was evaluated at presentation and on repeat testing, with and without the inclusion of a 20% delta change in cardiac troponin concentration.^25^ In a secondary analysis, we evaluated the diagnostic performance for type 1 or type 2 myocardial infarction. Readmission with type 1 myocardial infarction, cardiac death, and all-cause death were reported at 1 year.

### Statistical Analysis

Baseline characteristics across eGFR categories were presented as mean (SD) or median (interquartile range) for normally distributed and nonnormally distributed variables, respectively, and as proportions for categorical variables. A high-sensitivity cardiac troponin I concentration of <5 ng/L at presentation conferred a NPV of 99.6% across the whole population for the primary outcome.^7^ This threshold was evaluated in those patients with normal and impaired renal function. Because we expected the NPV to be close to 100%, we estimated the proportion by sampling from a binomial likelihood with a Jeffreys prior (β-distribution shape parameters both equal to 0.5), because intervals produced using this approach have good coverage for proportions close to 0 or 1.^26^ Survival free from type 1 myocardial infarction or cardiac death above and below the threshold of 5 ng/L was compared. Multivariable Cox proportional hazards models were performed to evaluate the association of eGFR and outcomes. Statistical analyses were performed using R, version 3.3.2.

## Results

### Patient Characteristics

Of 4739 patients enrolled, 4726 patients (99.7%) had at least 1 measure of serum creatinine (Figure I in the online-only Data Supplement). Of these, 904 patients (19%) had renal impairment with an eGFR <60mL/min/1.73m^2^ (Table 1). Most patients had moderate impairment (85%) with 15% having severe impairment (<30mL/min/1.73m^2^), and 13 (0.3%) patients receiving dialysis (12 on hemodialysis, 1 on peritoneal dialysis) (Table II in the online-only Data Supplement). Renal function was 2-fold higher in those with normal renal function (eGFR 91±18 versus 47±8 mL/min/1.73m^2^; *P*<0.001).

**Table 1.**
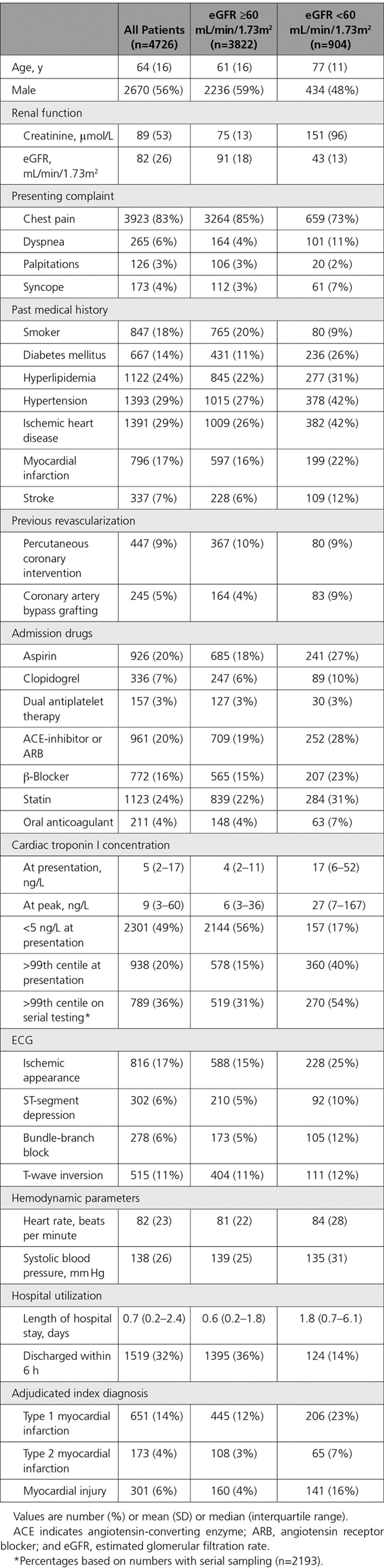
Cohort Characteristics in All Patients and Stratified by Renal Function

In comparison with patients with normal renal function, those with renal impairment were older and more likely to be women (Table 1). Baseline cardiovascular risk factors, including diabetes mellitus and hypertension, and established ischemic heart disease, as well, were more prevalent in those with renal impairment. Prescriptions of antiplatelet agents, blockers of the renin-angiotensin system, and statins were more frequent in this group. However, smoking was less common. Although more patients with renal impairment had previously undergone coronary artery bypass grafting, the rate of percutaneous coronary intervention was similar to those with normal renal function.

### Renal Impairment and Risk Stratification With Low Cardiac Troponin Concentrations at Presentation

**Figure 1.**
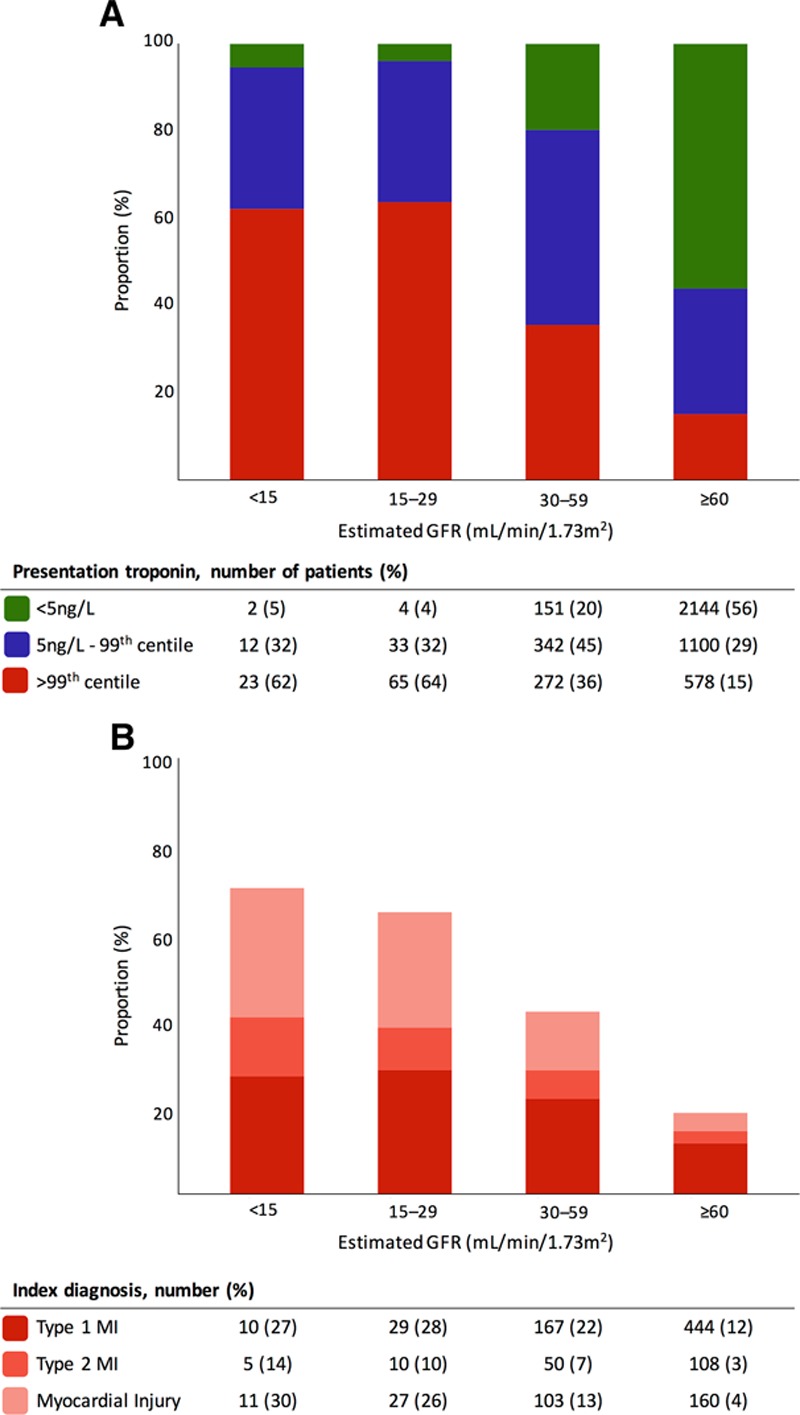
**Cardiac troponin I concentration at presentation stratified by renal function (A) and adjudicated index diagnosis >99th centile stratified by renal function (B).** GFR indicates glomerular filtration rate; and MI, myocardial infarction.

A cardiac troponin concentration of <5 ng/L at presentation identified 17% (157/904) of patients with and 56% (2144/3822) of patients without renal impairment as low risk (Figure 1A). The primary outcome of index type 1 myocardial infarction, or type 1 myocardial infarction or cardiac death within 30 days occurred in 1% (2/157) of those with renal impairment and in 0.3% (7/2144) of those with normal renal function. The NPV and sensitivity for the primary outcome was 98.4% (95% CI, 96.0%–99.7%) and 98.9% (95% CI, 97.5%–99.9%) in patients with renal impairment, in comparison with 99.7% (95% CI, 99.4%–99.9%) and 98.4% (95% CI, 97.2%–99.4%) in those without (Table 2). Performance was similar when the primary outcome was extended to include all myocardial infarction (type 1 and type 2) (Table III in the online-only Data Supplement). In our sensitivity analysis, the NPV and sensitivity at the limit of detection were similar to the risk stratification threshold, but this threshold identified only 19/904 (2%) of patients with renal impairment as low risk, in comparison with 628/3822 (16%) of those with normal renal function (Table IV in the online-only Data Supplement).

**Table 2.**
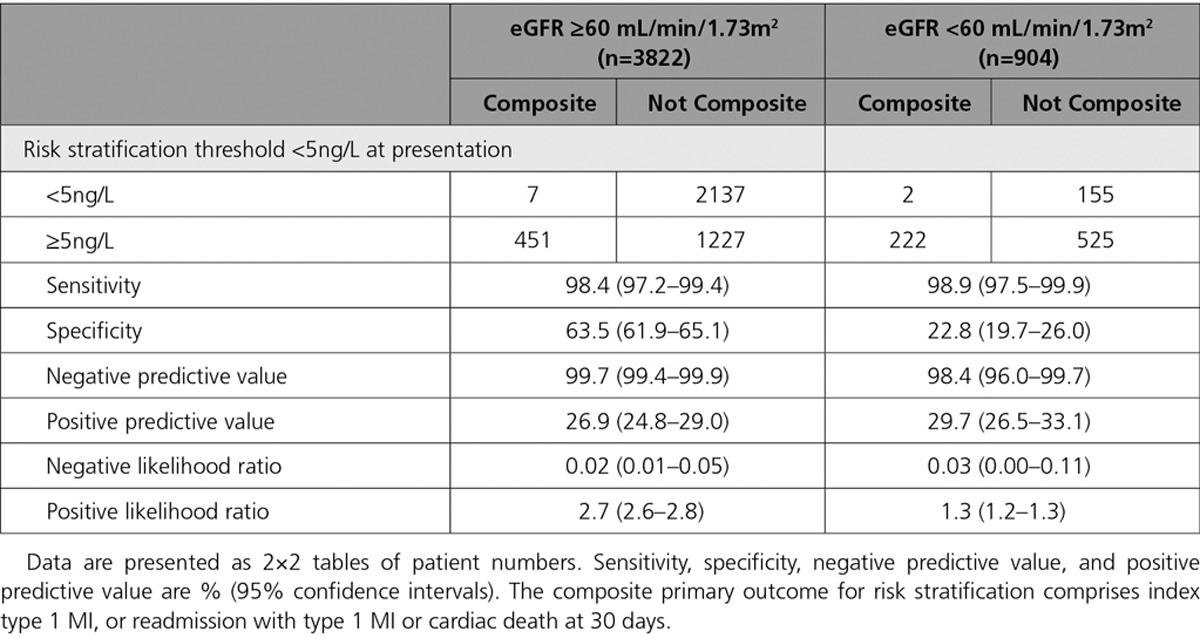
Performance of Risk Stratification Thresholds Stratified by Renal Function

### Renal Impairment and the Diagnosis of Myocardial Infarction

Cardiac troponin concentrations were >99th centile at presentation in 40% (360/904) of patients with and 15% (578/3822) without renal impairment (Figure 1A). During the index presentation, the adjudicated diagnosis was type 1 myocardial infarction in 23% (206/904) of patients with renal impairment in comparison with 12% (445/3822) of those with normal renal function (Figure 1B). Similarly, an adjudicated diagnosis of type 2 myocardial infarction was more frequent in patients with renal impairment occurring in 7% (65/904), in comparison with 3% (108/3822) of those with normal renal function. In those with renal impairment, the diagnosis of type 1 myocardial infarction occurred in 22% (167/765) of patients with moderate, 28% (29/102) with severe, and 27% (10/37) of patients with end-stage renal impairment. The diagnosis of type 2 myocardial infarction occurred in 7% (50/765) of patients with moderate, 10% (10/102) with severe, and 14% (5/37) of patients with end-stage renal impairment.

At the 99th centile, the PPV and specificity for an index type 1 myocardial infarction were lower in patients with renal impairment (50.0%; 95% CI, 45.2%–54.8% and 70.9%; 95% CI, 67.5%–74.2%, respectively), in comparison with those without (62.4%; 95% CI, 58.8%–65.9% and 92.1%; 95% CI, 91.2%–93.0%, respectively) (Table 3). The area under the curve for type 1 myocardial infarction was 0.95 (95% CI, 0.93–0.96) in patients without renal impairment in comparison with 0.82 (95% CI, 0.78–0.86) in those with renal impairment. The PPV and specificity were similar in patients with renal impairment stratified by age or sex, but were lower in those >65 years old and in women without renal impairment (Table V in the online-only Data Supplement).

**Table 3.**
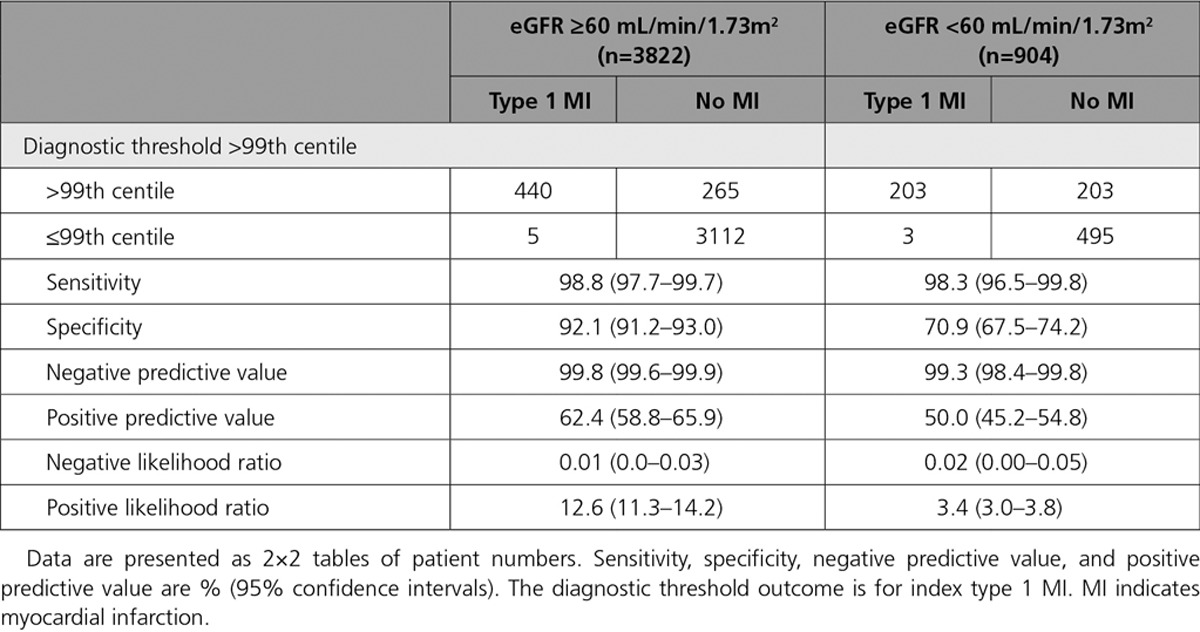
Performance of Diagnostic Thresholds Stratified by Renal Function

Sensitivity was similar in patients with and without renal impairment, both at presentation and on retesting (Table V in the online-only Data Supplement). In those patients with serial sampling (2193/4726, 46%), combining the 99th centile with a 20% delta change increased specificity in those with renal impairment from 68.8% (95% CI, 63.8%–73.6%) to 78.1% (95% CI, 73.6%–82.4%), but reduced sensitivity from 97.8% (95% CI, 95.5%–99.7%) to 78.4% (95% CI, 72.0%–84.7%). In contrast, combining the 99th centile with a 20% delta change did not significantly improve specificity in patients without renal impairment (90.5%; 95% CI, 88.9%–92.1% with a delta versus 88.1%; 95% CI, 86.4%–89.8% without), but sensitivity was lower (81.8%; 95% CI, 77.8%–85.7% with a delta versus 98.5%; 95% CI, 97.2%–99.6% without).

For the diagnosis of type 1 or type 2 myocardial infarction performance improved in all patients, although the PPV and specificity remained lower in those with renal impairment (65.7%; 95% CI, 61.0%–70.3% and 78.0%; 95% CI, 74.8%–81.2%, respectively), in comparison with those with normal renal function (77.5%; 95% CI, 74.4%–80.5% and 95.2%; 95% CI, 94.4%–95.9%, respectively; Table VI in the online-only Data Supplement).

### Renal Impairment and Risk of Type 1 Myocardial Infarction or Cardiac Death at 1 Year

Using Cox regression modeling, adjusted for age, sex, diabetes mellitus, hypertension, established ischemic heart disease, and cardiac troponin, we confirmed that renal impairment was an independent risk factor for subsequent type 1 myocardial infarction or cardiac death in the year following presentation (Figure II in the online-only Data Supplement). This model independently confirmed that the risk of major cardiac events increased once the eGFR fell to <60mL/min/1.73m^2^.

### Cardiac Troponin Risk Stratifies Patients With Normal and Impaired Renal Function

**Figure 2.**
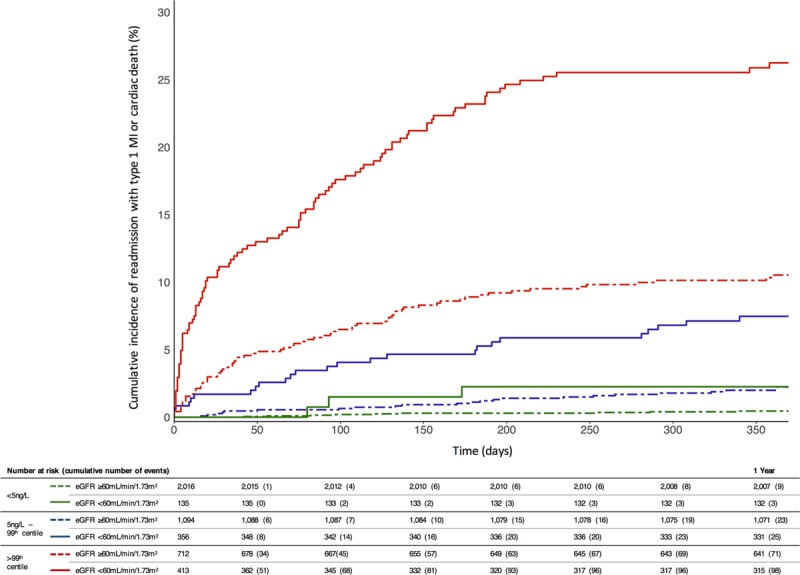
**Cumulative incidence plot for a composite of readmission with type 1 myocardial infarction or cardiac death at 1 year stratified by troponin and renal function.** Across all patients, the risk of readmission with type 1 myocardial infarction or cardiac death increased with cardiac troponin (HR, 1.15; 95% CI, 1.07–1.25 per doubling; *P*<0.001) and eGFR (HR, 1.28; 95% CI, 1.15–1.41 per fall of 10 mL/min/1.73m^2^; *P*<0.001) after adjustment for age, sex, established ischemic heart disease, diabetes mellitus, hypertension, and a cardiac troponin: eGFR interaction term (see Table VII in the online-only Data Supplement for detailed model). CI indicates confidence interval; eGFR, estimated glomerular filtration rate; HR, hazard ratio; and MI, myocardial infarction.

For all patients, subsequent type 1 myocardial infarction or cardiac death at 1 year was more frequent with increasing cardiac troponin concentrations (Figure 2). Irrespective of the index diagnosis, in patients with any cardiac troponin concentration >99th centile the 1-year risk of subsequent type 1 myocardial infarction or cardiac death was greater in those with renal impairment than in those without (24% versus 10%; adjusted hazard ratio, 2.19 [95% CI, 1.54–3.11]; Table 4). Where cardiac troponin concentrations remained <5 ng/L on serial testing, cardiac events at 1 year were uncommon in patients with or without renal impairment (2% versus 0.4%; adjusted hazard ratio, 2.49; 95% CI, 0.58–10.71). Patients with renal impairment and cardiac troponin concentrations ≥5 ng/L but ≤99th centile had an event rate similar to those with normal renal function and cardiac troponin concentrations >99th centile (7% and 10%, respectively).

**Table 4.**
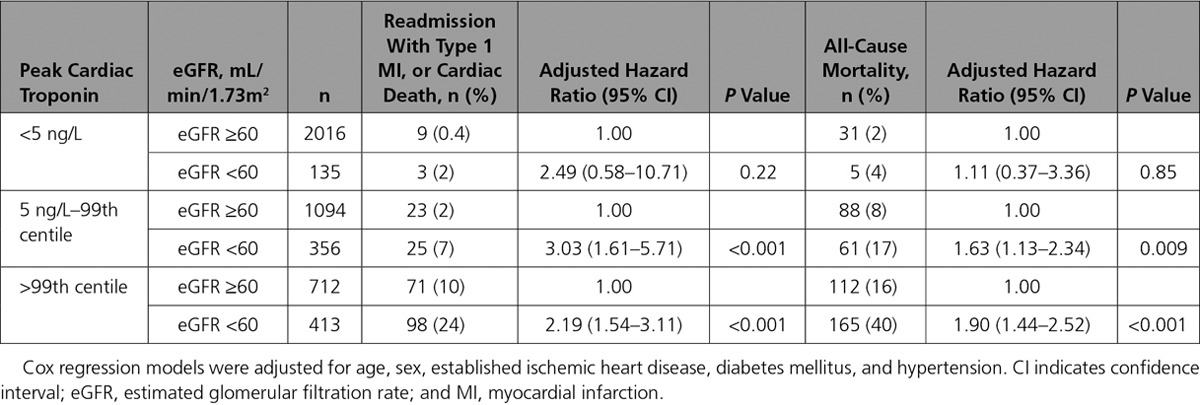
Outcomes at 1 Year Stratified by Peak Cardiac Troponin Concentration and Renal Function

**Figure 3.**
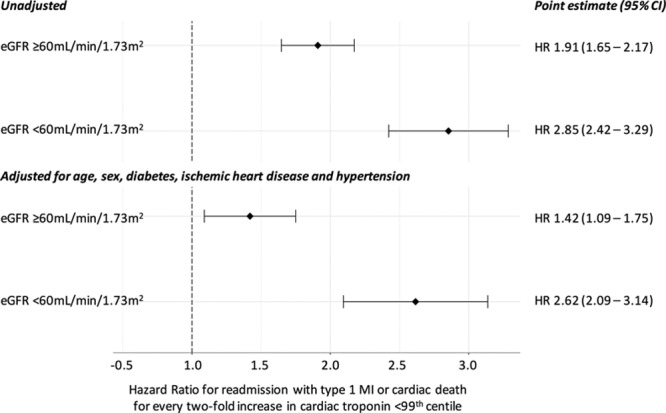
**Cox regression models for readmission with type 1 myocardial infarction or cardiac death at 1 year per 2-fold increase in cardiac troponin concentration below the 99th centile.** CI indicates confidence interval; eGFR, estimated glomerular filtration rate; HR, hazard ratio; and MI, myocardial infarction.

Increasing cardiac troponin concentrations below the 99th centile were associated with a greater risk of subsequent type 1 myocardial infarction or cardiac death at 1 year, after adjustment for age, sex, established ischemic heart disease, diabetes mellitus, or hypertension (Figure 3). For every doubling of cardiac troponin concentration in those with renal impairment, risk of cardiac events increased more than 2-fold (hazard ratio, 2.62; 95% CI, 2.09–3.14) in comparison with a more modest increase in those with normal renal function (hazard ratio, 1.42; 95% CI, 1.09–1.75).

## Discussion

In this large, prospective cohort of consecutive patients, we assessed the utility of high-sensitivity cardiac troponin I testing to risk stratify and diagnose patients with suspected acute coronary syndrome who have renal impairment. We make several relevant observations for clinical practice. First, high-sensitivity cardiac troponin I concentrations <5 ng/L at presentation identified patients who were at low risk of myocardial infarction or cardiac death at 30 days regardless of renal function. However, <1 in 5 with renal impairment were identified as low risk, in comparison with more than half of those with normal renal function. Second, patients with renal impairment were more than twice as likely to have cardiac troponin concentrations >99th centile. Here, the PPV and specificity for type 1 myocardial infarction were lower than for those with normal renal function. However, 1 in 4 patients with renal impairment had an index diagnosis of type 1 myocardial infarction, and this remained the most common cause of elevated cardiac troponin concentrations in this group. Third, irrespective of the diagnosis, patients with cardiac troponin concentrations >99th centile and renal impairment had a 2-fold greater risk of subsequent type 1 myocardial infarction or cardiac death at 1 year. Finally, while increasing cardiac troponin concentrations <99th centile independently predicted subsequent cardiac events in all patients, for an equivalent increase in concentration, patients with renal impairment had twice the risk of a major cardiac event as those with normal renal function. Together these findings suggest that high-sensitivity cardiac troponin testing may improve the risk stratification of patients with suspected acute coronary syndrome and renal impairment by identifying both low-risk patients who could avoid hospitalization and high-risk patients who may benefit from further investigation and therapies.

Our study has a number of strengths. We prospectively identified all consecutive patients without selection presenting to both secondary and tertiary care hospitals, including patients admitted out-of-hours. Moreover, we included patients with all degrees of renal impairment including those with a severe reduction in eGFR or on dialysis, a group often excluded from diagnostic studies.^27,28^ Thus, we consider our findings to be both representative and generalizable.

Our findings in those patients with renal impairment and cardiac troponin concentrations <5 ng/L at presentation support the use of this approach for risk stratification across all patients. Our analysis was conservative, using a composite primary outcome that included cardiac events at 30 days. The diagnostic sensitivity of 98.9% in patients with renal impairment would be considered by most as evidence that this approach could be safely applied in practice. The use of this threshold would potentially miss 2 index events in 904 consecutive patients with renal impairment. Indeed, a cardiac troponin concentration <5 ng/L was associated with just a 2% risk of subsequent type 1 myocardial infarction or cardiac death in these patients at 1 year. While this approach to risk stratification appears safe, it is clearly less effective in those with renal impairment, identifying <1 in 5 patients as low risk in comparison with more than half of those with normal renal function. This observation likely reflects the higher prevalence of shared risk factors, preexisting or unrecognized cardiovascular disease, or direct cardiac injury by uremic proteins in patients with renal impairment.

Our data add to those of others who have evaluated the effectiveness of high-sensitivity cardiac troponins for the diagnosis of myocardial infarction.^16,28–31^ However, the majority of studies have included selected patients, not examined those with renal impairment separately, or have adjudicated outcomes by using contemporary assays. A strength of our analysis is that all diagnoses and outcomes were adjudicated by using the high-sensitivity assay. In keeping with previous studies, the specificity of high-sensitivity cardiac troponin at the 99th centile for a diagnosis of type 1 myocardial infarction was reduced in patients with renal impairment.^26^ When considering the clinical utility of this diagnostic test, a reduction in PPV from 60% to 47% is arguably modest. One approach to improve specificity would be to use higher diagnostic thresholds in those with renal impairment.^28,32^ However, this approach assumes that all grades of renal impairment are equivalent, and implies to clinicians that small increases in cardiac troponin concentration are less important or are unrelated to cardiovascular risk in patients with renal impairment. Our analysis from a cohort of consecutive patients with the full spectrum of renal function, demonstrates that patients with renal impairment who have elevated cardiac troponin concentrations have twice the risk of a major cardiac event as those with normal renal function. Furthermore, increases in cardiac troponin concentration within the normal reference range are a stronger predictor of cardiac events in patients with renal impairment, likely reflecting the higher burden of risk factors and cardiovascular disease in these patients. So although higher thresholds might improve diagnostic accuracy, there is a risk this approach may be falsely reassuring to clinicians.

Renal impairment is associated with poor outcomes. In a recent study, renal impairment, defined as any reduction in eGFR, was responsible for 4% of all deaths worldwide, where more than half were a consequence of cardiovascular disease.^33^ Our data confirm this excess risk in patients with suspected acute coronary syndrome, but importantly suggest that cardiac troponin may be used to improve the risk stratification of these patients. It is interesting to note that we have shown that patients with renal impairment in whom myocardial infarction has been excluded have an incident cardiovascular risk similar to those with myocardial injury or infarction who have normal renal function. Furthermore, we demonstrate a 2-fold increase in cardiovascular risk for every doubling of cardiac troponin in patients with renal impairment in comparison with those with normal renal function. This observation supports those who suggest that elevations in cardiac troponin concentrations in kidney disease reflect underlying cardiovascular disease, rather than impaired renal clearance.^12^

There is often a disparity in the use of common, evidence-based treatments or interventions in patients with renal impairment.^34^ Far from this therapeutic nihilism in the face of poor outcomes,^34^ high-sensitivity cardiac troponin could be used to improve the targeting of therapy to this vulnerable and high-risk group of patients. Here, <30% of patients with renal impairment were prescribed aspirin, a blocker of the renin-angiotensin system or a statin. Whether we can improve outcomes in this high-risk group of patients through increasing the use of these preventative therapies should be the urgent focus of future clinical studies.

We recognize some limitations to our study. Only 15% of patients with reduced eGFR had severe renal impairment, with just 13 patients (1%) on dialysis, so caution must be taken when interpreting these results in this group of patients. The study was conducted in a predominantly white population (93%); therefore, further evaluation in more diverse populations would be of interest. Similarly, our findings are limited to a single high-sensitivity cardiac troponin I assay and cannot be extrapolated to other assays.^32^ Furthermore, clinical management decisions were made using a contemporary assay, and, therefore, it is possible that a small number of patients only identified by the high-sensitivity assay may have experienced worse outcomes because myocardial injury was not recognized by their treating clinician. However, this limitation affected all patients, and therefore does not impact the validity of our comparison between those with and without renal impairment. We classified patients based on a single estimate of renal function, and it is unclear whether those with renal impairment had acute kidney injury or chronic kidney disease. Both are associated with future cardiovascular risk.^1,35^ However, our approach is consistent with clinical practice where renal function and cardiac troponin are measured concurrently and treatment decisions are largely based on measures of renal function at the time of presentation.

In conclusion, high-sensitivity cardiac troponin I may improve the risk stratification of patients with suspected acute coronary syndrome who have renal impairment by identifying low-risk patients who could avoid hospitalization, and high-risk patients who may benefit from further investigation and therapies.

## Sources of Funding

Dr Anand is supported by a Research Fellowship from Chest Heart and Stroke Scotland (15/A163). Dr Chapman is supported by a Project Grant and a Fellowship (PG/15/51/31596, FS/16/75/32533) from the British Heart Foundation (BHF). Dr Halbesma is supported by a BHF Intermediate Basic Science Research Fellowship (FS/16/36/32205). Prof Newby is the recipient of a Wellcome Trust Senior Investigator Award (WT103782AIA). Profs Mills and Newby are supported by the Butler Senior Research Fellowship (FS/16/14/32023) and Chair (CH/09/002) awards from the BHF. Dr Dhaun is supported by a BHF Intermediate Clinical Research Fellowship (FS/13/30/29994).

## Disclosures

Drs Anand, Shah, and Chapman have received honoraria from Abbott Diagnostics. Prof Mills has acted as a consultant for Abbott Diagnostics, Beckman-Coulter, Roche, and Singulex. All other authors report no conflicts.

## Supplementary Material
