# Supplementary material for: High-Sensitivity Cardiac Troponin and the Risk Stratification of Patients With Renal Impairment Presenting With Suspected Acute Coronary Syndrome
Source: Circulation. 2018 Jan 29;137(5):425–35. doi: 10.1161/CIRCULATIONAHA.117.030320 (PMC5793996; doi:10.1161/CIRCULATIONAHA.117.030320)

SUPPLEMENTARY MATERIAL

High-sensitivity cardiac troponin and the risk stratification of patients with renal impairment presenting with suspected acute coronary syndrome

Eve Miller-Hodges, MBChB, PhD,[* 1, 2] Atul Anand, MBChB,[* 1] Anoop S V Shah, MBChB, PhD, [1] Andrew R Chapman, MBChB, [1] Peter Gallacher, MBChB,[1, 2] Kuan Ken Lee, MBChB,[1] Tariq Farrah, MBChB,[1, 2] Nynke Halbesma, MBChB,[3] James P Blackmur, MBChB,[1] David E. Newby, MBChB, PhD,[1] Nicholas L Mills, MBChB, PhD,[1] Neeraj Dhaun, MBChB, PhD[1,2]

[*] *These authors contributed equally*

Risk stratification in ACS with renal impairment

[1] University/British Heart Foundation Centre for Cardiovascular Science, University of Edinburgh, Queen's Medical Research Institute, 47 Little France Crescent, Edinburgh, EH16 4TJ, UK

[2] Department of Renal Medicine, Royal Infirmary of Edinburgh, 51 Little France Crescent, Edinburgh, EH16 4SA, UK

[3] Usher Institute of Population Health Sciences and Informatics, University of Edinburgh, Nine Edinburgh BioQuarter, 9 Little France Road Edinburgh EH16 4UX, UK

Correspondence: Dr Neeraj Dhaun
Queen's Medical Research Institute
47 Little France Crescent
Edinburgh
EH16 4TJ
(+44)-131-242-6786
bean.dhaun@ed.ac.uk

Pages: 12 **Supplementary Tables:** 7 **Supplementary Figures:** 2

Supplementary Table 1: Diagnostic Criteria

Type 1 myocardial infarction	Myocardial necrosis (any high-sensitivity cardiac troponin I concentration above sex-specific 99th centile upper reference limit) with rise and or fall in hs-cTnI concentration where serial testing was available AND symptoms OR signs of myocardial ischemia
Type 2 myocardial infarction	Myocardial necrosis (high-sensitivity cardiac troponin I concentration above sex-specific 99th centile upper reference limit) with rise and or fall in high-sensitivity cardiac troponin I concentration where serial testing was available AND symptoms OR signs of myocardial ischemia AND evidence of increased oxygen demand (e.g. tachyarrhythmia, hypertrophy) or reduced supply (e.g. hypotension or anemia) in context of alternative clinical diagnosis
Myocardial injury	Myocardial necrosis (high-sensitivity cardiac troponin I concentration above sex-specific 99th centile upper reference limit) without symptoms OR signs of myocardial ischemia in context of alternative clinical diagnosis

Supplementary Table 2

	**eGFR <15 mL/min/1.73m²** *(n=37)*	**eGFR 15-29 mL/min/1.73m²** *(n=102)*	**eGFR 30–59 mL/min/1.73m²** *(n=765)*	**eGFR ≥60 mL/min/1.73m²** *(n=3,822)*
Age (years)	68 (15)	78 (11)	77 (10)	61 (16)
Male	26 (70%)	55 (54%)	353 (46%)	2,236 (59%)
Renal Function				
Creatinine (μmol/L)	528 (185)	224 (49)	123 (26)	75 (13)
eGFR (mL/min/1.73m²)	10 (3)	24 (4)	47 (8)	91 (18)
Presenting complaint				
Chest pain	21 (57%)	60 (59%)	578 (76%)	3,264 (85%)
Dyspnea	4 (11%)	22 (22%)	75 (10%)	164 (4%)
Palpitations	1 (3%)	1 (1%)	18 (2%)	106 (3%)
Syncope	3 (8%)	9 (9%)	49 (6%)	112 (3%)
Past medical history				
Smoker	5 (14%)	5 (5%)	72 (9%)	765 (20%)
Diabetes mellitus	13 (35%)	32 (31%)	191 (25%)	431 (11%)
Hyperlipidemia	8 (22%)	29 (28%)	240 (31%)	845 (22%)
Hypertension	21 (57%)	35 (34%)	322 (42%)	1,015 (27%)
Ischemic heart disease	14 (38%)	44 (43%)	324 (42%)	1,009 (26%)
Myocardial infarction	8 (22%)	27 (26%)	164 (21%)	597 (16%)
Stroke	3 (8%)	17 (17%)	89 (12%)	228 (6%)
Previous revascularization				
Percutaneous intervention	0 (0%)	5 (5%)	75 (10%)	367 (10%)
Coronary artery bypass grafting	6 (16%)	6 (6%)	71 (9%)	164 (4%)
Admission drugs				
Aspirin	8 (22%)	23 (23%)	210 (27%)	685 (18%)
Clopidogrel	3 (8%)	9 (9%)	77 (10%)	247 (6%)
Dual antiplatelet therapy	2 (5%)	1 (1%)	27 (4%)	127 (2%)
ACE-inhibitor or ARB	4 (11%)	21 (21%)	227 (30%)	709 (19%)
Beta-blocker	6 (16%)	21 (21%)	180 (24%)	565 (15%)
Statin	6 (16%)	30 (29%)	248 (32%)	839 (22%)
Oral anticoagulant	1 (3%)	5 (5%)	57 (7%)	148 (4%)
Cardiac troponin I concentration				
At presentation (ng/L)	49 (14–151)	41 (18–122)	14 (6–42)	4 (2–11)
At peak (ng/L)	242 (58–833)	54 (20–1491)	22 (7–115)	6 (3–36)
<5ng/L at presentation	2 (5%)	4 (4%)	151 (20%)	2144 (56%)
>99th centile at presentation	23 (62%)	65 (64%)	272 (36%)	578 (15%)
Electrocardiogram				
Ischemic appearance	13 (35%)	30 (29%)	185 (24%)	588 (15%)

ST-segment depression	8 (22%)	13 (13%)	71 (9%)	210 (5%)
Bundle branch block	3 (8%)	17 (17%)	85 (11%)	173 (5%)
T-wave inversion	5 (14%)	12 (12%)	94 (12%)	404 (11%)
Hemodynamic parameters				
Heart rate (beats per minute)	87 (27)	88 (27)	83 (28)	81 (22)
Systolic BP (mmHg)	144 (39)	127 (37)	136 (30)	139 (25)
Hospital utilization				
Length of hospital stay (days)	2.7 (0.9–8.2)	5.1 (1.2–11.8)	1.3 (0.3–5.0)	0.6 (0.2–1.8)
Discharged within 6 hours	6 (16%)	2 (2%)	116 (15%)	1395 (36%)
Adjudicated index diagnosis				
Type 1 myocardial infarction	10 (27%)	29 (28%)	167 (22%)	445 (12%)
Type 2 myocardial infarction	5 (14%)	10 (10%)	50 (7%)	108 (3%)
Myocardial injury	11 (30%)	27 (26%)	103 (13%)	160 (4%)

Values are number (%) or mean (SD) or median (interquartile range)
Abbreviations: eGFR = estimated glomerular filtration rate; ACE = angiotensin converting enzyme; ARB = angiotensin receptor blocker; BP = blood pressure

Supplementary Table 3: Performance of the risk stratification threshold (<5ng/L) for index type 1 myocardial infarction or readmission with type 1 myocardial infarction or cardiac death within 30 days, and index type 1 or type 2 myocardial infarction or readmission with type 1 or 2 myocardial infarction or cardiac death within 30 days

	**eGFR ≥60mL/min/1.73m²** *(n=3,822)*		**eGFR <60mL/min/1.73m²** *(n=904)*	
Composite including only type 1 MI				
	Composite	Not Composite	Composite	Not Composite
<5ng/L	7	2137	2	155
≥5ng/L	451	1227	222	525
**Sensitivity**	**98.4 (97.2–99.4)**		**98.9 (97.5–99.9)**	
Specificity	63.5 (61.9–65.1)		22.8 (19.7–26.0)	
**Negative predictive value**	**99.7 (99.4–99.9)**		**98.4 (96.0–99.7)**	
Positive predictive value	26.9 (24.8–29.0)		29.7 (26.5–33.1)	
Composite including type 1 or type 2 MI				
	Composite	Not Composite	Composite	Not Composite
<5ng/L	10	2134	2	155
≥5ng/L	555	1123	285	462
**Sensitivity**	**98.1 (97.0–99.2)**		**99.1 (98.1–99.9)**	
Specificity	65.5 (63.9–67.1)		25.2 (21.8–28.6)	
**Negative predictive value**	**99.5 (99.2–99.8)**		**98.4 (96.0–99.7)**	
Positive predictive value	33.1 (30.9–35.4)		38.2 (34.7–41.7)	

Data presented as 2x2 tables of patient numbers. Sensitivity, specificity, negative predictive value and positive predictive value are % (95% confidence intervals).

Supplementary Table 4: Diagnostic performance of the limit of detection (1.2ng/L) on presentation as a risk stratification threshold in patients with and without renal impairment

	**eGFR ≥60mL/min/1.73m^2** *(n=3,822)*		**eGFR <60mL/min/1.73m^2** *(n=904)*	
Risk stratification threshold <1.9ng/L (LoD)				
	Composite	Not Composite	Composite	Not Composite
<1.2ng/L	3	625	0	19
≥1.2ng/L	455	2739	224	661
**Sensitivity**	**99.2 (98.4–99.9)**		**99.8 (99.1–100.0)**	
Specificity	18.6 (17.3–19.9)		2.9 (1.7–4.1)	
**Negative predictive value**	**99.4 (98.7–99.9)**		**97.5 (90.5–100.0)**	
Positive predictive value	14.3 (13.1–15.5)		25.3 (22.5–28.3)	

Data presented as 2x2 tables of patient numbers. Sensitivity, specificity, negative predictive value and positive predictive value are % (95% confidence intervals). The composite primary outcome for risk stratification comprises index type 1 myocardial infarction, or readmission with type 1 myocardial infarction or cardiac death at 30 days.

Supplementary Table 5: Sub-group analysis of the diagnostic performance of the 99th centile for a diagnosis of type 1 myocardial infarction

	Type 1 MI (%)	TP	TN	FP	FN	Sensitivity	Specificity	NPV	PPV
**All Patients (n=4,726)**									
eGFR≥60ml/min/1.73m^2	445 (12)	440	3112	265	5	98.8 (97.7-99.7)	92.1 (91.2-93.0)	99.8 (99.6-99.9)	62.4 (58.8-65.9)
eGFR<60ml/min/1.73m^2	206 (23)	203	495	203	3	98.3 (96.5-99.8)	70.9 (67.5-74.2)	99.3 (98.4-99.8)	50.0 (45.2-54.8)
**Males (n=2,670)**									
eGFR≥60ml/min/1.73m^2	284 (13)	280	1843	109	4	98.4 (97.0-99.7)	94.4 (93.4–95.4)	99.8 (99.5-99.9)	71.9 (67.4–76.3)
eGFR<60ml/min/1.73m^2	100 (23)	98	252	82	2	97.5 (94.5-99.8)	75.4 (70.7-79.9)	99.0 (97.5-99.8)	54.4 (47.1-61.6)
**Females (n=2,056)**									
eGFR≥60ml/min/1.73m^2	161 (10)	160	1269	156	1	99.1 (97.6-100.0)	89.0 (87.4-90.6)	99.9 (99.6-100.0)	50.6 (45.1-56.1)
eGFR<60ml/min/1.73m^2	106 (23)	105	243	121	1	98.6 (96.4-100.0)	66.7 (61.9-71.5)	99.4 (98.1-100.0)	46.5 (40.0-53.0)
**≥65 years old (n=2,411)**									
eGFR≥60ml/min/1.73m^2	247 (15)	243	1186	190	4	98.2 (96.5-99.6)	86.2 (84.3-88.0)	99.6 (99.2-99.9)	56.1 (51.4-60.7)
eGFR<60ml/min/1.73m^2	183 (23)	180	419	186	3	98.1 (96.1-99.7)	69.2 (65.5-72.9)	99.2 (98.1-99.8)	49.2 (44.1-54.3)
**<65 years old (n=2,315)**									
eGFR≥60ml/min/1.73m^2	198 (9)	197	1926	75	1	99.2 (98.0-100.0)	96.2 (95.4-97.0)	99.9 (99.8-100.0)	72.3 (66.9-77.5)
eGFR<60ml/min/1.73m^2	23 (20)	23	76	17	0	97.9 (92.1-100.0)	81.4 (73.5-88.9)	99.4 (97.5-100.0)	57.3 (42.1-71.9)

**Serial sampling on presentation (n=2,193)**									
eGFR≥60ml/min/1.73m^2	359 (21)	263	1210	122	96	73.2 (68.6-77.7)	90.8 (89.2-92.3)	92.6 (91.1-94.0)	68.3 (63.5-72.8)
eGFR<60ml/min/1.73m^2	159 (32)	121	247	96	38	75.9 (69.3-82.4)	71.9 (67.2-76.6)	86.5 (82.4-90.2)	55.7 (49.1-62.3)
**Serial sampling on repeat testing (n=2,193)**									
eGFR≥60ml/min/1.73m^2	359 (21)	354	1174	158	5	98.5 (97.2-99.6)	88.1 (86.4-89.8)	99.5 (99.1-99.8)	69.1 (65.0-73.0)
eGFR<60ml/min/1.73m^2	159 (32)	156	236	107	3	97.8 (95.5-99.7)	68.8 (63.8-73.6)	98.5 (96.7-99.6)	59.3 (53.3-65.1)
**>99th centile and ≥20% delta change (n=2,193)**									
eGFR≥60ml/min/1.73m^2	359 (21)	294	1206	126	65	81.8 (77.8-85.7)	90.5 (88.9-92.1)	94.9 (93.6-96.0)	70.0 (65.5-74.2)
eGFR<60ml/min/1.73m^2	159 (32)	125	268	75	34	78.4 (72.0-84.7)	78.1 (73.6-82.4)	88.6 (84.8-91.9)	62.4 (55.7-69.0)

Diagnostic threshold >99th centile tested against an outcome of index type 1 myocardial infarction. TP = true positives; TN = true negatives; FP = false positives; FN = false negatives; NPV = negative predictive value; PPV = positive predictive value. Metrics provided are % (95% confidence intervals).

Supplementary Table 6: Performance of the diagnostic threshold (99th centile) for index type 1 myocardial infarction and index type 1 or type 2 myocardial infarction

	eGFR ≥60mL/min/1.73m^2 *(n=3,822)*		eGFR <60mL/min/1.73m^2 *(n=904)*	
Type 1 MI				
	Type 1 MI	No MI	Type 1 MI	No MI
>99th centile	440	265	203	203
≤99th centile	5	3112	3	495
Sensitivity	98.8 (97.7–99.7)		98.3 (96.5–99.8)	
**Specificity**	**92.1 (91.2–93.0)**		**70.9 (67.5–74.2)**	
Negative predictive value	99.8 (99.6–99.9)		99.3 (98.4–99.8)	
**Positive predictive value**	**62.4 (58.8–65.9)**		**50.0 (45.2–54.8)**	
Type 1 or type 2 MI				
	Type 1 or 2 MI	No MI	Type 1 or 2 MI	No MI
>99th centile	547	158	267	139
≤99th centile	6	3111	4	494
Sensitivity	98.8 (97.9–99.6)		98.3 (96.8–99.6)	
**Specificity**	**95.2 (94.4–95.9)**		**78.0 (74.8–81.2)**	
Negative predictive value	99.8 (99.6–99.9)		99.1 (98.1–99.7)	
**Positive predictive value**	**77.5 (74.4–80.5)**		**65.7 (61.0–70.3)**	

Data presented as 2x2 tables of patient numbers. Sensitivity, specificity, negative predictive value and positive predictive value are % (95% confidence intervals).

Supplementary Table 7: Cox proportional hazard models for predictors of readmission with type 1 myocardial infarction or cardiac death up to 1 year (229 events)

Variable	Model 1	Model 2	Model 3	Model 4	Model 5
Age *per* 10 year increase	2.02*** (1.81-2.26)	1.74*** (1.57-1.95)	1.47*** (1.30-1.66)	1.44*** (1.26-1.63)	1.44*** (1.26-1.63)
Male sex	1.56** (1.19-2.04)	1.39* (1.06-1.82)	1.52** (1.16-1.98)	1.58** (1.18-2.11)	1.57** (1.18-2.10)
Cardiac troponin *per* doubling		1.22*** (1.19-1.26)	1.20*** (1.17-1.24)	1.20*** (1.16-1.23)	1.15*** (1.07-1.25)
eGFR *per* fall of 10mL/min/1.73m^2			1.22*** (1.15-1.29)	1.23*** (1.15-1.30)	1.28*** (1.15-1.41)
Previous IHD				1.32 (1.00-1.75)	1.32 (1.00-1.75)
Diabetes				1.36 (0.99-1.87)	1.37 (1.00-1.88)
Hypertension				0.86 (0.65-1.14)	0.87 (0.66-1.16)
Cardiac troponin: eGFR interaction					1.00 (1.00-1.00)

No significant difference between Model 4 and Model 5 by ANOVA (p=0.31)
***p<0.001; **p<0.01; *p<0.05

Supplementary Figure 1: Study CONSORT diagram

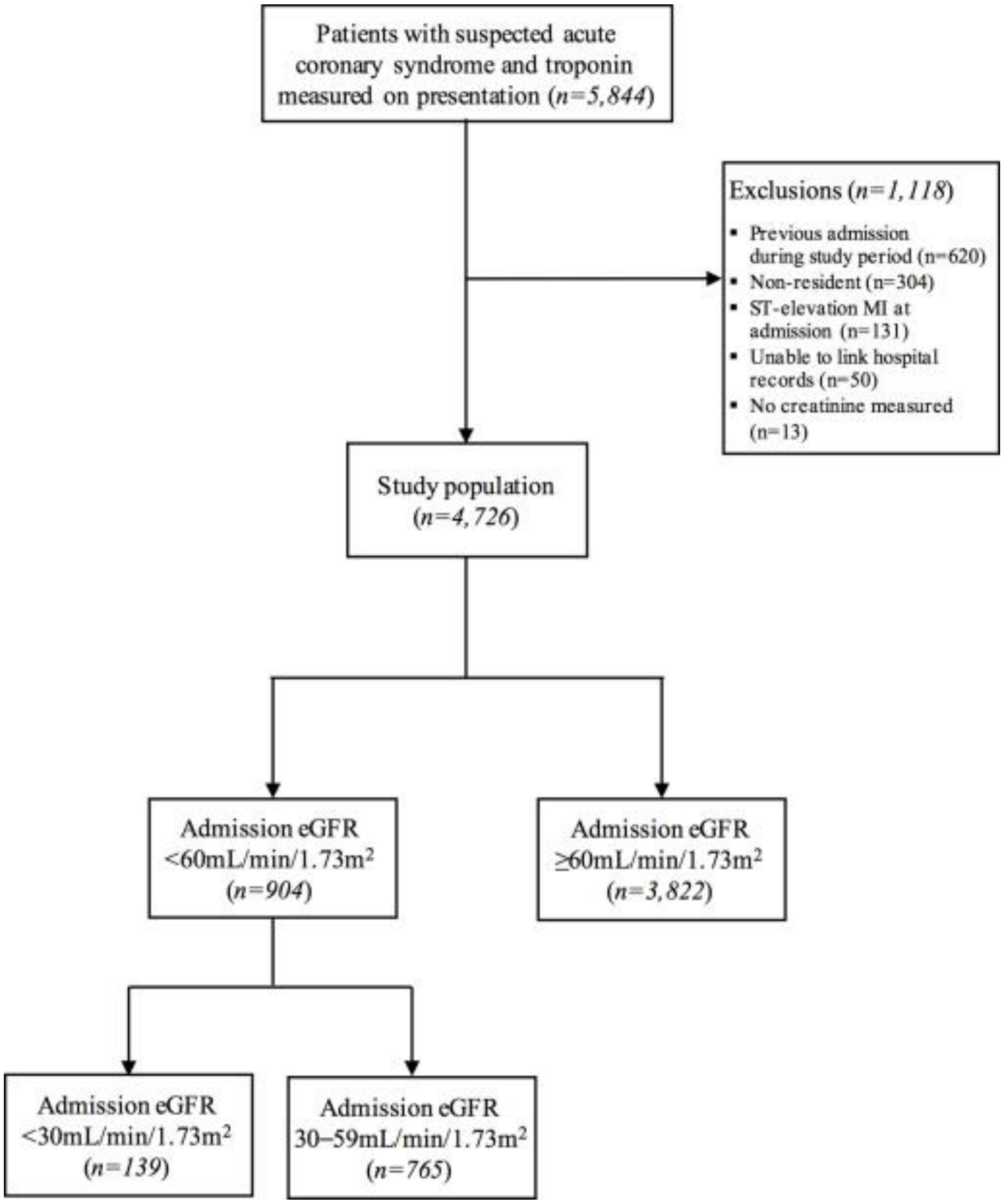

Supplementary Figure 2: Cox regression modelling of the relationship between estimated glomerular filtration rate and the risk of subsequent type 1 myocardial infarction or cardiac death

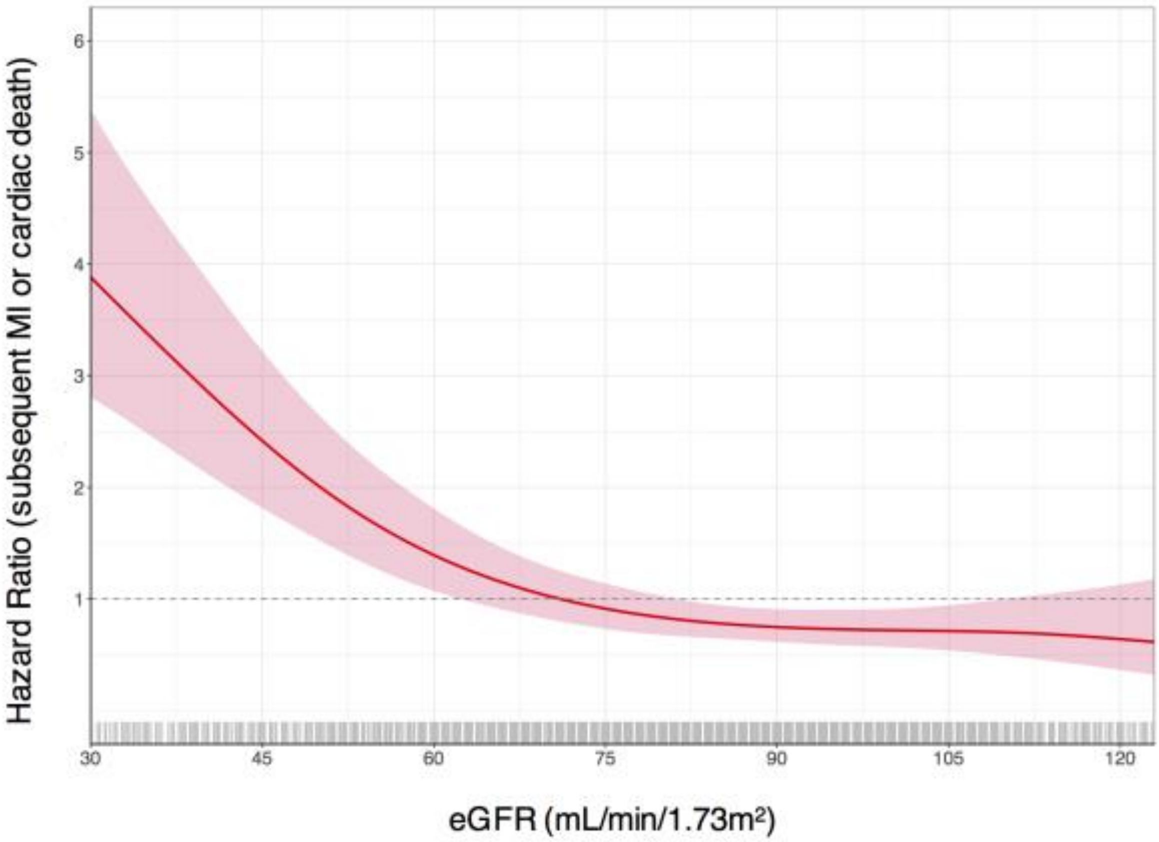

Supplement: Supplementary file 1 [file cir-137-425-s001.pdf]
